# Oncological resection, myasthenia gravis and staging as prognostic factors in thymic tumours: a Chilean case series

**DOI:** 10.3332/ecancer.2021.1201

**Published:** 2021-03-09

**Authors:** Patricio Salas, Maria Eliana Solovera, Felipe Bannura, Matias Muñoz-Medel, Miguel Cordova-Delgado, Cesar Sanchez, Carolina Ibañez, Marcelo Garrido, Erica Koch, Francisco Acevedo, Sebastian Mondaca, Bruno Nervi, Jorge Madrid, Jose Peña, Mauricio P Pinto, José Valbuena, Hector Galindo

**Affiliations:** 1 Thoracic Surgery Section, Division of Surgery, Faculty of Medicine, Pontificia Universidad Catolica de Chile, Diagonal Paraguay 319, Santiago 8330032, Chile; 2 Department of Hematology and Oncology, Faculty of Medicine, Pontificia Universidad Catolica de Chile, Diagonal Paraguay 319, Santiago 8330032, Chile; 3 Department of Pathology, Faculty of Medicine, Pontificia Universidad Catolica de Chile, Diagonal Paraguay 319, Santiago 8330032, Chile

**Keywords:** thymic tumour, thymoma, myasthenia gravis, staging

## Abstract

**Background:**

Thymic epithelial tumours are rare and highly heterogeneous. Reports from the United States suggest an overall incidence of 0.15 per 100,000/year. In contrast, the incidence of these tumours in Latin America is largely unknown and reports are scarce, somewhat limited to case reports.

**Methods:**

Herein, we report a series of 38 thymic tumours from a single institution, retrospectively incorporated into this study. Patient characteristics and outcomes including age, sex, stage, paraneoplastic syndromes, treatment regimens and the date of decease were obtained from medical records.

**Results:**

Most cases in our series were females and young age (<50 years old) and early stage by Masaoka-Koga or the Moran staging systems. Also, a 34% of patients had myasthenia gravis (MG). Next, we analysed overall survival rates in our series and found that the quality of surgery (R0, R1 or R2), MG status and staging (Masaoka-Koga, Moran or TNM) were prognostic factors. Finally, we compared our data to larger thymic tumour series.

**Conclusions:**

Overall, our study confirms complete surgical resection as the standard, most effective treatment for thymic epithelial tumours. Also, the Masaoka-Koga staging system remains as a reliable prognostic factor but also the Moran staging system should be considered for thymomas.

## Introduction

Thymic neoplasms are a rare and highly heterogeneous group of tumours. Reports from the United States indicate an overall incidence of 0.15 per 100,000/year [1]. Histologically, thymic tumours can be classified as thymomas, thymic carcinomas or neuroendocrine thymic tumours. These tumours are frequently associated with paraneoplastic diseases, among these myasthenia gravis (MG) is by far the most commonly reported. Indeed, most studies demonstrate that MG prevalence on thymoma patients ranges from 30% to 50% [2]. Although there is still no consensus on an official staging classification system for thymic malignancies [3], the system originally developed by Masaoka *et al* [4] and subsequently modified by Koga *et al* [5] remains as the most widely accepted. This system (hereafter called Masaoka-Koga) establishes four stages based on the local extent of the disease [6]. More recently, Moran *et al* [7] proposed a staging system based on pathological characteristics and specifically focused on thymomas and their invasiveness. Similarly, a number of histological classifications have been proposed for thymic tumours over the last decades; however, the World Health Organization histological classification system is the most accepted [8, 9]. Regarding treatment, the current gold standard for patients is complete resection surgery sometimes accompanied by radiation or chemotherapy [10].

As pointed above, thymic tumours are rare; however, their incidence in Latin America is largely undefined and the evidence is scarce and scattered, somewhat limited to case reports. Herein, we report a Chilean series of 38 thymic epithelial tumours from a single institution, most cases correspond to young patients (<50-year-old) and early stage thymomas. We also report survival rates and compare our results to other published thymoma series.

## Materials and methods

### Patients and ethics approval

A total of 38 thymoma/thymic carcinoma patients were retrospectively incorporated into this study. Patients were diagnosed between December 1996 and April 2018. This study was approved by the Internal Review Board and Ethics committee at the Pontificia Universidad Catolica de Chile (approval #171004005 dated on 7 November 2017). A waiver of consent was granted for deceased patients.

### Eligibility criteria and collected data

Inclusion criteria for this study were: diagnosis of thymic epithelial tumour (either thymoma or thymic carcinoma), adult individuals (>18-year-old) at the time of diagnosis, availability of medical records including clinical management and follow-up and being able to understand, read and sign a written informed consent form. A minimum of 3 months of follow-up after surgical treatment was also considered as necessary for the incorporation of patients into this study. Collected data included: patient characteristics and outcomes such as age, sex, stage, paraneoplastic syndromes related to thymic malignancies, treatment regimens including chemotherapy, surgery and/or radiation therapy. The dates of decease were also obtained from medical records available at the Centro de Cancer in the Pontificia Universidad Catolica de Chile or the Red de Salud UC Christus in Santiago, Chile.

### Follow up, survival rates and statistics

Last date of follow-up was 1 August 2019. Overall survival (OS) was defined as the time between diagnosis and death by any cause. Survival curves were estimated using the Kaplan–Meier method. Univariate associations between patient sex, age, stage, surgical resection and survival were analysed by Log-rank test. Statistical significance was set at *p* < 0.05. Statistical analyses were performed using the R program software (version 3.5.1 R Development Core Team, R Foundation for Statistical Computing, Vienna, Austria), applying the ‘survival’ and ‘survminer’ packages.

## Results

### Patients’ characteristics

Demographic and basic patient characteristics are summarised in Table 1. Briefly, most patients were female, younger than 50-year-old and early stage by Masaoka-Koga (50% were stage I). Similarly, most thymomas in our series were categorised as stage 0–I (84%) according to the staging system described by Moran *et al* [7]. As expected, the most frequent paraneoplastic symptom was MG (34%). All patients had thymic resection surgery by sternotomy (84%), video-assisted thoracic surgery (13%) or by thoracotomy (8%). Patients’ subsets also received chemotherapy or radiation therapy, 37% or 21%, respectively.

### Survival rates and current thymoma series versus other cohorts

**Figure 1.**
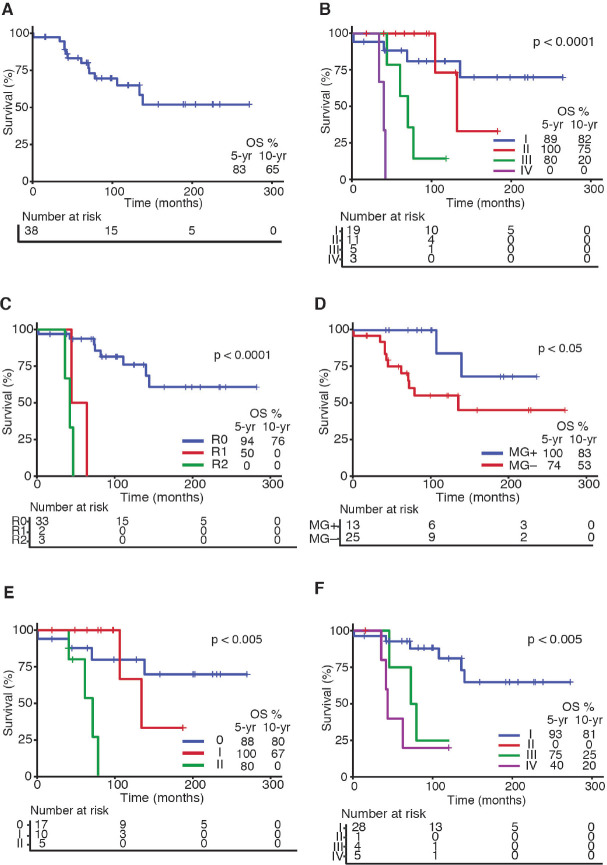
Survival curves in Chilean thymic tumour series. (a): OS for the entire series (*n* = 38). (b): OS by Masaoka-Koga staging system at presentation. (c): OS by status of surgical margins at oncological resection. (d): OS by presence or absence of MG at diagnosis. (e): OS by Moran staging at diagnosis. (f): OS by TNM staging at diagnosis. Abbreviations: OS, overall survival; yr, Year; MG, Myasthenia gravis.

OS rates for the entire series are shown in Figure 1a. As expected, tumour stage at initial diagnosis had a significant impact on OS (*p* < 0.0001, Figure 1b). Five-year OS was 89%, 100%, 80% and 0% for Masaoka stage I, II, III, IV, respectively. All patients underwent surgical resection with curative intent. Negative resection margins with pathologically confirmed (R0) were achieved in 33 (87%) patients. Otherwise, three (8%) and two (5%) patients had microscopic residual disease (R1) and gross residual disease (R2). Also as expected, patients with an optimal surgery (R0) had better OS versus suboptimal (R1 or R2) counterparts (*p* < 0.0001, Figure 1c). Five-year OS was 94%, 50% and 0% for R0, R1 and R2 resection, respectively. Patients that were MG+ had better OS rates versus MG− counterparts (*p* < 0.05, Figure 1d). Then, we analysed OS in the subset of thymomas (*n* = 32) in our series according to the Moran *et al* [7] staging system and found a significant impact on survival (Figure 1e; *p* < 0.005). Lastly, as shown in Table 2, we compared OS rates by Masaoka-Koga stage in our series against other previously published cohorts from Japan [4], Canada [11], the Netherlands [12] and China [13]. Patient follow-up and other symptoms are summarized in Supplementary Table S1.

## Discussion

Thymic tumours are infrequent neoplasms. Epidemiological studies in the US indicate an incidence of 0.15 cases per 100,000/year [1]. In contrast, thymic tumour incidence in Latin America is largely unknown. Furthermore, regional studies on thymic tumours/thymomas are extremely scarce. Two retrospective single-institution studies in Mexico City have reported 64 [14] and 25 [15] patients, respectively. The first study reports a 10-year database in which 54.7% were males and average age (at diagnosis) was 51.4 years. 28% of patients were MG+. The second study reports clinical characteristics of patients recruited in the period January 2005–December 2016, although these data are unpublished, they were presented at the International Association for the Study of Lung Cancer 18th World Conference on Lung Cancer [15]. In Chile, a recent study reported survival rates and basic characteristics of 65 thymic tumour patients [16]; however, this report does not provide information on long-term OS rates (5- or 10-year). In general, thymoma series demonstrate a predominance of females over males, and diagnosis at an early stage. Accordingly, our study found that most patients (63%) were female and early stage (50% were stage I). As expected, OS rates in our study correlated with tumour stage by Masaoka-Koga. This is also in line with published larger thymoma series (Table 2) that demonstrate lower 5- and 10-year OS at increasing tumour stages. Over the last century, a variety of staging systems have been proposed for thymic tumours [3]; however, the Masaoka-Koga staging system remains as the preferred choice in the literature. Within this context, a recent study by Weissferdt *et al* [17] proposes a simplified staging system for thymomas, originally described by Moran *et al* [7]. Authors used a large cohort (*n* = 1,470 cases) and demonstrate a significant correlation with clinical outcomes [17]. Accordingly, in our data, we found a significant correlation to OS using this staging system; however, this was limited to the subset of thymomas (Figure 1e). Also as expected, the most frequent paraneoplastic symptom was MG (34%). Interestingly, our data suggest that MG+ patients had better OS versus MG− counterparts (Figure 1d). Indeed, previous studies have described a better OS in MG+ thymomas [18]. However, subsequent stage-adjusted studies indicated no differences in survival between MG+ and MG− thymomas [8, 19, 20]. More recently, a large retrospective study found that MG+ had a slight protective effect on OS among thymoma patients. Once again, this association could not be confirmed by a multivariate analysis. Similarly, the abovementioned retrospective study in Mexico found a trend towards better OS rates in MG+ that did not reach statistical significance. Although a potential link between MG+ and better OS in these patients remains controversial, authors have speculated that the correlation between MG+ and early Masaoka-Koga stages could explain this association [9]. Furthermore, the same study also demonstrated that a higher Masaoka-Koga stage correlated with non-MG status. Evidently, our findings warrant further investigation and should be confirmed by larger series.

## Conclusion

Overall, our study confirms complete surgical resection as the standard, most effective treatment for thymic tumours. Similarly, the Masaoka-Koga staging system remains as a reliable prognostic factor for these tumours. In the same way, the Moran staging system for thymomas demonstrates a better stratification for this subset of thymic tumours, especially at 10-years and further. Unexpectedly, our data report a statistically significant difference in OS regarding MG status, with better rates of OS at 5 and 10 years for MG+ patients. Finally, this study has a number of limitations: first and foremost, the number of cases is relatively small compared to other published series (Table 2), this is partially explained by the total Chilean population (17.5 million) and the low incidence rates of these tumours. Secondly, as pointed out, our data were retrospectively obtained at a single institution which may represent a registration bias that may limit the scope of our findings.

## Availability of data and materials

The data used and analysed on this study are available from the corresponding author on reasonabe request.

### Contributions

PS and MES conceived the present study. MES and MMM collected the data. MCD, MMM and MPP analysed and interpreted the data. MPP drafted the manuscript. PS, MES, FB, CS, CI, MG, EK, FA, SM, BN, JM, JP, JV and HG critically revised the manuscript. All authors read and approved the final version of the manuscript.

## Ethics declaration

### Ethics approval and consent to participate

Study and ethics approval were obtained by the Internal Review Board and Ethics committee at the Pontificia Universidad Catolica de Chile (approval #171004005 dated on 7 November, 2017).

## Funding statement

None.

## Competing interests

The authors declare that they have no competing interests.

## Figures and Tables

**Supplementary Table S1.** Onset symptoms, follow-up and survival of study population (*n* = 38).

	*n*	%
**Presenting symptoms**		
Arterial hypertension	7	18
Diabetes mellitus	1	3
Sjögren syndrome	2	5
Dyslipidaemia	5	13
SLE	1	3
Cancer^a^	6	16
Chest pain	6	16
Hypothyroidism	4	11
Hyperparathyroidism	1	3
Diplopia	3	8
SVC syndrome	1	3
Asymptomatic	5	13
**Follow-up and survival**		
Alive	25	66
Median follow-up (range), months	83 (1–271)	

SLE, Systemic lupus erythematosus; SVC, Superior vena cava

a One case of thymoma had a concomitant finding of early-stage lung adenocarcinoma

**Table 1.** Demographic, pathologic and treatment management characteristics of study population (*n*=38).

Characteristics	*n*	%		Characteristics	*n*	%
**Age at diagnosis**				**WHO histologic classification**		
25-49 y	18	47		A	5	13
50-70 y	16	42		AB	5	13
>70 y	4	11		B1	6	16
**Sex**				B2	9	24
Male	14	37		B3	6	16
Female	24	63		C	7	18
**Stage at diagnosis (Masaoka-Koga)**				**Paraneoplastic symptoms**		
I	19	50		Myasthenia gravis	13	34
IIa	8	21		Pure red cell aplasia	2	5
IIb	3	8		Nephrotic syndrome	2	5
III	5	13		Agranulocytosis	1	3
IVa	3	8		Surgical margins		
**Stage at diagnosis (Moran)**				R0	33	87
0	16	59		R1	2	5
I	7	26		R2	3	8
II	3	11		**Resection of regional lymph nodes**		
III	1	4		Yes	21	55
**Primary tumor size (AJCC 8^th^ Edition)**				No	14	37
T1a	29	76		NA	3	8
T2	1	3		**Chemotherapy**		
T3	7	18		No	24	63
T4	1	3		Yes	5	13
**Regional lymph nodes (AJCC 8^th^ Edition)**				NA	9	24
N0	37	97		**Radiation therapy**		
N1	1	3		No	22	58
**Distant metastasis (AJCC 8^th^ Edition)**				Yes	8	21
M0	34	89		NA	8	21
M1a	4	11		**Thymic epithelial tumor histotype**		
**TNM stage at diagnosis (AJCC 8^th^ Edition)**				Thymoma	31	82
I	28	74		Thymic carcinoma	6	16
II	1	3		Mixed†	1	3
IIIa	4	11		**Surgery type**		
IVa	5	13		Sternotomy	32	84
				VATS	5	13
				Thoracotomy	1	3

*VATS*, video-assisted thoracic surgery; *NA*, not available.

† One case evidenced both thymoma and thymic carcinoma cells during histologic analysis.

**Table 2.** Survival rates comparison of thymoma series by Masaoka-Koga staging system.

Stage	Chilean thymomas				Masaoka *et al* [4]		Mariano *et al* [11]		d de Jong *et al* [12]	Zhu *et al* [13]
	n	5-yr OS%	10-yr OS%		5-yr OS%	10-yr OS%	5-yr OS%	10-yr OS%	5-yr OS%	5-yr OS%
I	19	89	82		96	67	93	93	83	96
II	11	100	75		86	60	89	87	88	89
III	5	80	20		70	58	75	62	57	59
IV	3	0	0		50	0	43	29	56	50

yr, Year; OS, Overall survival
